# Glymphatic system clearance and Alzheimer’s disease risk: a CSF proteome-wide study

**DOI:** 10.1186/s13195-024-01612-7

**Published:** 2025-01-31

**Authors:** Natalia Cullell, Giovanni Caruana, Andrea Elias-Mas, Ariane Delgado-Sanchez, Cristina Artero, Maria Teresa Buongiorno, Marta Almería, Nicola J. Ray, Sonia A. L. Correa, Jerzy Krupinski

**Affiliations:** 1 Fundació per a Docència I Recerca, MútuaTerrassa, Terrassa, Barcelona, Spain; 2 Department of Neurology, F.Ass. MútuaTerrassa, Terrassa, Barcelona, Spain; 3 Department of Radiology, F.Ass. MútuaTerrassa, Terrassa, Barcelona, Spain; 4 Institute for Research and Innovation Parc Taulí (I3PT), Sabadell, Spain; 5 Genetics Doctorate Program, Universitat de Barcelona (UB), Barcelona, Spain; 6 Department of Psychology, Brooks Building, Faculty of Science and Education, Manchester Metropolitan University, Manchester, UK; 7 Department of Life Sciences John Dalton Building, Faculty of Science and Engineering, Manchester Metropolitan University, Manchester, UK

**Keywords:** Alzheimer’s disease, Glymphatic system, Proteomics, Mild cognitive impairment, MRI, Inflammation

## Abstract

**Background:**

The emerging evidence of the role of the glymphatic system (GS) in Alzheimer’s disease (AD) provides new opportunities for intervention from the earliest stages of the disease. The aim of the study is to evaluate the efficacy of GS in AD to identify new disease biomarkers.

**Methods:**

We performed a two-stage proteomic study to evaluate the GS health using intravenous gadolinium-based contrast agent (GBCA) with serial T1 3T magnetic resonance imaging (MRI) in individuals with amnestic mild cognitive impairment (aMCI). In Stage 1 (evaluated in the Cohort 1 of aMCI participants (*n* = 11)), we correlated the levels of 7K cerebrospinal fluid (CSF) proteins (estimated by SOMAscan) with GS health in 78 Freesurfer-segmented brain regions of interest (ROIs).

**Results:**

A total of seven different proteins were significantly associated with GS health (*p*-value < 6.4 × 10^–4^). The stronger correlations were identified for NSUN6, GRAAK, OLFML3, ACTN2, RUXF, SHPS1 and TIM-4. A pathway enrichment analysis revealed that the proteins associated with GS health were mainly implicated in neurodegenerative processes, immunity and inflammation. In Stage 2, we validated these proteomic results in a new cohort of aMCI participants (with and without evidence of AD pathology in CSF (aMCI(-) and aMCI/AD( +); *n* = 22 and 7, respectively) and healthy controls (*n* = 10). Proteomic prediction models were generated in each ROI. These were compared with demographic-only models for identifying participants with aMCI(-) and aMCI/AD( +) vs controls. This analysis was repeated to determine if the models could identify those with aMCI/AD( +) from both aMCI(-) and controls. The proteomic models were found to outperform the demographic-only models.

**Conclusions:**

Our study identifies proteins linked with GS health and involved the immune system in aMCI participants.

**Supplementary Information:**

The online version contains supplementary material available at 10.1186/s13195-024-01612-7.

## Background

Alzheimer’s disease (AD) is a highly prevalent neurodegenerative disease affecting over 50 million people worldwide [1] and is the most common cause of dementia [2]. Accumulation of extracellular soluble amyloid-β plaques and intracellular neurofibrillary tangles of phosphorylated tau proteins is a hallmark of AD [3]. In addition, sustained brain immune responses—which are linked to amyloid-β and tau accumulation – have emerged as contributors to disease progression [4].

It is predicted that by 2050 there will be 130 million people with AD worldwide [5]. Preventative strategies are critically needed but require a better characterization of risk factors and the detection of early stages of the disease. Participants with AD positive biomarkers are often divided into three stages: the preclinical stage, characterized by normal cognitive ability; the prodromal stage, characterized by mild cognitive impairment (MCI); and the dementia stage, with functional impairment [6]. It is highly likely that future disease-modifying strategies will depend on interventions applied at the earliest stages of the disease in participants with MCI [2]. In this regard, the glymphatic system (GS) has emerged as a potential therapeutic target [7].

The GS is one route by which fluid and solutes are cleared from the brain [8], and its function is thought to be directly dependent on sleep [8]. This system consists of perivascular spaces (PVS) around arteries, arterioles and veins, covered by a network of vascular endfeet of astrocytes. The water channel aquaporin (AQP) 4 located in the endfeet of astrocytes is thought to be critical for GS function [8]. The cerebrospinal fluid (CSF) influx from subarachnoid space enters the brain via the PVS facilitated by AQP-4. CSF is then pushed via arterial pulsatility to move through the interstitium. After CSF-interstitial fluid (ISF) exchange, the fluid exits the brain via the PVS surrounding veins. The clearance of interstitial solutes from the brain then proceeds via meningeal and cervical lymphatic vessels [9]. This system may therefore be important for the clearance of soluble amyloid-β from the brain.

In AD, alterations in PVSs and the blood–brain barrier alter the function of the GS, leading to failure of protein clearance [7]. Neuroinflammation may also be responsible for GS dysfunction [10]. Immune cells reside in the brain, but peripheral immune cells can also enter it. When homeostasis is disrupted, they evoke a neuroinflammatory cascade linked with AD risk and prognosis. The meningeal vessels are relevant in the functioning of the GS, and they are immune active sites [11]. When the meningeal lymph vessels are dysregulated, the autoimmune response is activated within leptomeningies [12]. Recent studies produced new insights into the complex barrier properties of the arachnoid. It showed that arachnoid barrier cells form a double layer, with cell-to-cell contacts and the whole is sewn together with tricellular junctions. They identified four different expression profiles of fibroblast forming arachnoid, glued to the arachnoid membrane and acting as one sole impermeable barrier. However, after inducing inflammation in transgenic mice, the authors watched via live imaging as T cells crawled along the pia mater. Occasionally, a T cell would flatten and cross the membrane, suggesting these cells needed to find specific sites that allowed them to transmigrate into the brain [13, 14]. This finding is relevant as inner arachnoid harbors immune cells, which may increase in number with age and inflammation.

Recent work has used intravenously administered gadolinium-based contrast agent (GBCA) to study the human glymphatic system [8], allowing the evaluation of clearance dynamics. In our recent study [15], we have evaluated the clearance of the intravenously injected GBCA in the same cohort of participants. We demonstrated that glymphatic system function is associated with AD-related changes to sleep, cognition and core AD biomarker concentrations in CSF in a group of participants with early-stage AD. Specifically, in our cohort of patients with aMCI/AD, faster/more efficient GBCA clearance was associated with shorter sleep latency, more intact global cognitive performance and robust relationships with CSF AD biomarkers [15].

Proteomic studies are useful to identify potential biomarkers in disease. In AD, proteomic studies have identified several proteins in blood and CSF that are associated with the risk for AD, the rate of cognitive decline, as well as hippocampus atrophy [16–18]. Recently, changes in proteins related to autophagy, ubiquitination and sugar metabolism in CSF have been found to be differentially expressed in people with AD compared to controls [19]. It is useful therefore to identify relationships between proteomic markers and GS function in the progression of AD.

Given that our cohort of participants exhibit glymphatic dysregulations associated with cognitive function (as seen in our previous results [15]), the primary objective of this study was to identify proteins associated with GS function in patients with aMCI-AD. This information is crucial to identify target proteins as possible GS health biomarkers. By linking GS proteomics with AD, we aimed to demonstrate that proteomic alterations associated with GS health are not only linked to changes in brain clearance rate but also correlate with clinically relevant AD phenotypes. Therefore, targeting GS may directly impact AD risk and prognosis. To achieve this, we conducted a proteome-wide analysis to investigate proteins linked to glymphatic system clearance activity in CSF.

## Methods

### Experimental design

A prospective, pilot study of out participants from the Cognition and Behaviour Unit at the Department of Neurology from the Hospital Universitari MútuaTerrassa (HUMT).

**Fig. 1:**
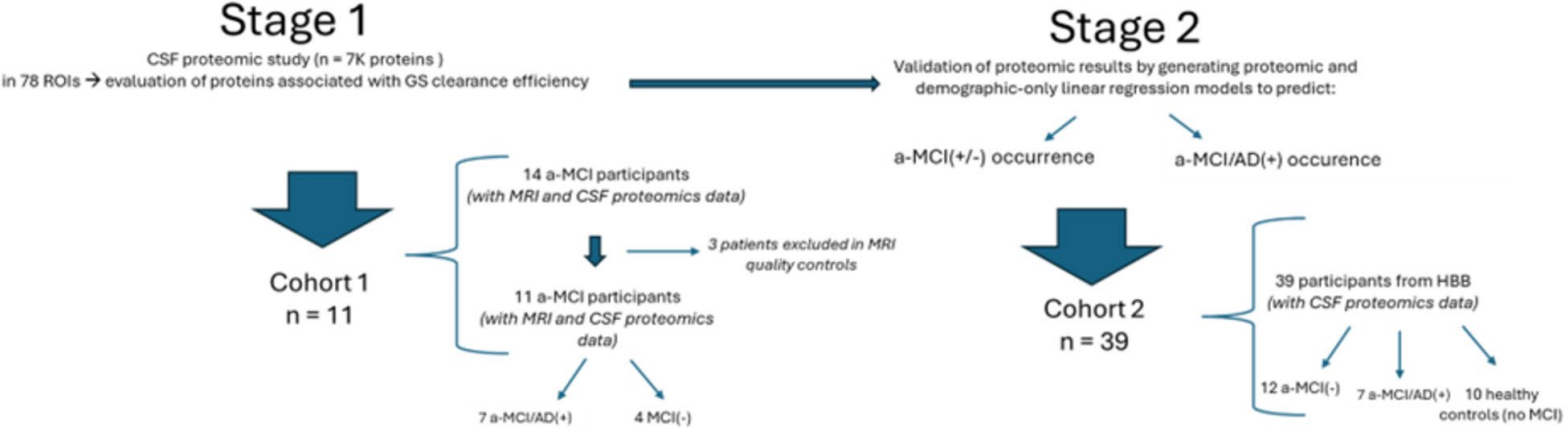
Description of the study workflow and cohorts. aMCI: amnestic mild cognitive impairment; GS: Glymphatic system. aMCI/AD( +): Participants with aMCI and positive CSF AD biomarkers. AMCI/AD( +) was considered when participants had Aβ1-42/Aβ1-40 < 0.068 plus at least two other positive biomarkers from Aβ1-42 < 638 pg/mL; total-tau > 404 pg/mL; p-tau 181 > 52.1 pg/mL; total-tau/ Aβ42 > 0.784

The study had two stages and was carried in two separate cohorts of participants (Fig. 1). In Stage 1, participants from Cohort 1 were studied. This cohort included participants with amnestic MCI (aMCI) according to DSM-5 diagnostic criteria [20] with imaging of GS health and CSF proteomics data. This cohort included 11 aMCI participants (seven with positive CSF AD biomarkers: aMCI/AD( +) and four with negative CSF AD biomarkers: aMCI(-)). Initially we screened 14 participants in Cohort 1, but 3 participants were excluded after MRI quality control checks. In Stage 1, we performed a proteomic study to evaluate the association of CSF proteins with GS health.

In Stage 2, we validated the proteomic associations and evaluated their predictive value in aMCI in an independent cohort (Cohort 2), consisting of 39 participants: 7 aMCI/AD( +), 22 aMCI(-), and 10 healthy controls (Fig. 1). Healthy controls were individuals without neurological disease who had CSF collected during anesthesia or emergency department procedures.

In the main cohort (Cohort 1), the participants with mild to moderate cognitive impairment were prospectively included based on the following criteria: male and female aged between 65 and 75 years; minimum reading and writing capacity to be able to perform the cognitive impairment tests, scored of at least 0.5 in the memory domain in the Clinical Dementia Rating (CDR). Objective cognitive performance was assessed using the MMSE and the delayed memory index of the Repeatable Battery for the Assessment of Neuropsychological Status (RBANS). To assess for evidence of AD pathological features, AD biomarkers were examined in participants CSF using the Lumipulse essay kits from Fujirebio (Fujirebio Inc. Europe, Gent, Belgium): Aβ40, Aβ42, ratio Aβ42/Aβ40, total tau, phosphorylated tau at threonine 181 (p-tau) and ratio tau/Aβ42. We determined positivity of AD core biomarkers using local cut off values established based on Álvarez I et al. [21]. Specifically, MCI/AD( +) participants were considered thus participants with Aβ1-42/Aβ1-40 < 0.068 plus at least two others from Aβ1-42 < 638 pg/mL; total-tau > 404 pg/mL; p-tau 181 > 52.1 pg/mL; total-tau/ Aβ42 > 0.784. The participants were also required to have a positive amyloid-PET to be considered MCI/AD( +).

Participants were excluded if they had previous diagnosis of other neurocognitive disorders, history of affective disorder or psychosis, attend at the time of inclusion to a regular cognitive training, take psychotropic or other medications that affect cognition (except stable hypnotic medication in the 4 weeks prior to inclusion), have history of cerebrovascular accident, transient ischemic attack or traumatic brain injury or any condition that, in the opinion of the clinician/investigator, interferes with compliance with the study procedures.

### MRI acquisition and analyses of signal intensity

#### MRI acquisition

MRI scans were acquired using a 3T MR scanner (Phillips Ingenia Elition). A standardized MR protocol was used for the acquisition, comprising of high-resolution 3D T1-weighted magnetization-prepared rapid gradient echo (MPRAGE) imaging sequence for enhanced tissue contrast, with the following settings: TR = 6.8, TE = 3.1; TI = 606.4 ms; flip angle = 8. Voxel dimensions were 1 × 1x1mm and the acquisition time for each scan was 4:13 min.

Following a pre-contrast MRI scan, participants were administered with intravenous injection of a gadolinium-based contrast agent. After that, new images were taken after 4 h and 28 h.

#### MRI Preprocessing and analysis

We applied FreeSurfer software (version 7) (http://surfer.nmr.mgh.harvard.edu/) for the segmentation, parcellation, and registration of the three T1-weighted images (using the longitudinal recon-all pipeline). Using a hybrid watershed/surface deformation procedure [22], non-brain tissue is removed, followed by the segmentation of the subcortical white matter and deep gray matter structures (including the hippocampus, amygdala, caudate, putamen, pallidum, and ventricles) as well as cortex [23]. The MR images of each patient were used to create a median template, and the images were then registered to the template applying a rigid transformation [24]. The registrations were checked manually to correct any registration errors. The segmentations and parcellations produced by recon-all for each scan were transformed back into native T1 space to extract T1 signal intensity within each region. Next, we used T1 signal intensity within a reference region in the posterior part of the orbit to normalize each scan according to changes in the gray-scale due to automatic image scaling, which will be different for each time point.

GS health was measured as GS clearance efficiency based on the following formula: (1-(28 h-baseline)/4 h-baseline)/3, with higher values indicating better clearance.

Using a region of interest (ROI) approach, we examined whether the change in contrast uptake across the ROI’s (*n* = 78) was associated with GS health indexed by above mentioned formula. We removed three participants from the study because of lack of GBCA enrichment in the parenchyma in almost all the ROIs (final sample size with GBCA and proteomic data included in the analyses = 11 participants).

#### Proteomic analysis

7K proteins were studied in CSF samples from the Cohort 1 and 2 using the SomaScan platform from SomaLogic.

SOMAscan is based on aptamers (called SOMAmers: (Slow Off-rate Modified Aptamers)) which are modified DNA aptamers with high affinity and high specificity for their cognate analytes. Using a multiplex technology, it allows the detection of up to 7,000 proteins in 40ul of CSF.

After processing samples and obtaining the proteomic data, different quality controls were applied. First, the data was normalized using hybridation controls to reduce the variation from the readout steps. Then, to reduce technical variation, the data was normalized by the median signal across pooled calibrator replicates. Then, plate scale and calibration scale were used to adjust for overall signal intensity differences between runs and for SOMAmer reagent-specific assay differences between runs. Finally, Median signal normalization was performed using Adaptive Normalization by Maximum Likelihood. Protein concentrations were quantified as relative fluorescence units (RFU).

The expression values for each protein were log10 transformed before the statistical analyses. All the proteins that were “flagged” for any of the above-mentioned quality controls were excluded. We also excluded proteins with a variability between samples less than 1%.

#### Statistical analysis

To describe the characteristics of each cohort, we calculated the mean and standard deviation for numeric and parametric variables, and the median and interquartile range for numeric and non-parametric variables. To assess differences between groups (aMCI(-) and aMCI/AD( +)), we used the t-test.

We evaluated the specific protein RFU associated with GS clearance efficiency using Spearman correlations in each of the 78 ROIs using R. After False Discovery Rate (FDR) correction, all the proteins with a *p*-value < 6.4 × 10^–4^ were considered significant. The association of each protein with the clearance was evaluated in each of the 78 ROIs. To plot the results in a volcano plot, we merged the results from the different ROIs: for each evaluated protein, we considered only the results from the ROI with the top association. Protein upregulation and downregulation was considered when the log2 Fold Change was higher or lower (respectively) than 1 standard deviation of the mean.

#### Pathway enrichment analysis (Stage 1 analysis)

The pathway or set enrichment analysis provides with information about the pathways where the proteins associated with a trait belong. We used WebGestalt [25], with the method Gene Set Enrichment Analysis (GSEA) and the Panther database.

We considered as input for these analyses the full list of analyzed proteins and the Rho statistics from the correlation between proteins and GS clearance efficiency. We used two different enrichment analyses approaches: 1) “Single analysis”: A total of 78 independent enrichment analyses were performed, with one analysis corresponding to each result from a different ROI. The pathways identified across all 78 analyses were then pooled together, and only those with a false discovery rate (FDR)-corrected *p*-value < 0.05 were considered significant. If a pathway was found to be significant in more than one ROI, this information is recorded in the Fig. 3. 2) Composite Analysis: A single analysis was conducted, taking into account only the Rho statistics from the ROI showing the strongest association for each specific protein. Pathways were considered significant if they had an FDR-corrected *p*-value < 0.05.

#### Proteomic and demographic prediction models (Stage 2 analysis)

The Stage 2 was carried out in Cohort 2 (consisting of 39 participants, who did not take part in Stage 1) (Fig. 1).

With the objective to evaluate whether proteins associated with GS health were able to predict the risk of aMCI occurrence, we generated prediction models incorporating proteomic scores and considering two scenarios: all aMCI (*n* = 29 participants: 7 MCI( +) and 22 MCI(-)) vs healthy controls (*n* = 10) and aMCI/AD( +) (aMCI/AD( +) participants (*n* = 7) vs 10 healthy controls + 22 aMCI(-)).

The generation of the proteomic scores was done as follows: for each ROI, we considered all the proteins associated with GS clearance efficiency with a *p*-value < 6.4 × 10^–4^. Using *regsubsets* function from leaps package in R, we selected the best score with a maximum of 5 proteins. One score was generated for each ROI, thus we created 78 different proteomics scores (as there were 78 ROIs) and each of them included from 1 to 5 proteins (depending on the number of significant proteins associated with clearance in each ROI). The score was generated weighting the proteins levels by the Spearman correlation coefficient (for the association of each protein with the clearance efficiency). For better understanding, we show the calculation for a specific patient and ROI:

$$\mathrm{For}\;\mathrm{patient}\;1\;\mathrm{and}\;\mathrm{ROI}\;1:\;\mathrm{Rho}\;\mathrm{protein}1\;\ast\;\mathrm{levels}\;\mathrm{protein}1\:+\:\mathrm{Rho}\;\mathrm{protein}2\;\ast\;\mathrm{levels}\;\mathrm{protein}2\;\lbrack\dots\rbrack\:+\:\mathrm{Rho}\;\mathrm{proteinX}\;\ast\;\mathrm{levels}\;\mathrm{proteinX}.$$

Where X is the number of proteins selected for the score from the regsubset function (maximum of five).

All these calculations were repeated for each of the 78 ROIs and the 39 participants from the Cohort 2 included in this analysis.

Next, we generated proteomic general linear regression models to predict the occurrence of aMCI(+ and -) and aMCI/AD( +). We constructed 78 different general linear models (glm) integrating the proteomic scores with demographic information (sex and age): *proteomic models*. Each proteomic model was compared for the prediction of aMCI with a model including only demographic variables (sex and age): *demographic-only model*.

Thus, in the cohort 2, we generated a total of 78 proteomic ROI-specific models for the aMCI occurrence prediction (each one including one ROI specific proteomic score, sex and age) and a demographic model including only sex and age. We also generated 78 proteomic ROI-specific models in the cohort 2 for the aMCI/AD( +) occurrence prediction and one demographic model. Additionally, we created proteomic ROI-specific models and a demographic model for aMCI/AD( +) prediction in the cohort 2 and the models performance was tested in the cohort 2. This approach was not followed for the prediction of aMCI(+ and -) because the cohort 1 did not includ healthy controls.

We evaluated the performance of the models assessing the model discrimination with the ROC curve (AUC). The specificity and sensitivity of each model was assessed with the Caret package after computing the best thresholds.

## Results

### Stage 1

#### Clinical and demographic outcomes

All participants in Cohort 1 were aMCI, *N* = 7 and *N* = 4 with and without evidence of AD pathology respectively (aMCI(+ and -)). They were evaluated for CSF AD biomarkers, neurocognitive function and overnight sleep quality (via polysomnography) See Table 1 for a summary of these characteristics.

**Table 1:** Cohort 1 description

		**aMCI/AD ( +); ** ***n*** **= 7**	**aMCI (-); ** ***n*** **= 4**	***P*** **-value**
Demographic	Age; mean (SD)	72.5 (2.38)	72 (7.25)	NS
	Sex (% Female)	6 (85.7%)	2 (50%)	NS
Biomarkers (mean)	Amyloid 1:42 (SD)	643.83 (214.25)	830.25 (235.89)	*p* < 0.005
	Tau (SD)	552.16 (232.85)	329.75 (106.15)	NS
	Ratio amyloid 1:42/1:40 (SD)	0.046 (0.01)	0.076 (0.003)	*p* < 0,05
	Ratio tau/amyloid 1:42 (SD)	0.90 (0.46)	0.40 (0.09)	p < 0.05
Cognition (median)	MMSE (IQR)	19 (10)	28 (0.5)	*p* < 0.05
	RBANS (IQR)	48 (9)	84 (5.75)	*p* < 0.05
	CDR (IQR)	0.5 (0.25)	0.5 (0)	NS

*MMSE* Mini-Mental State Examination, *RBANS* Repeatable Battery for the Assessment of Neuropsychological Status, *CDR* Clinical Dementia Rating, *SD* Standard deviation, *IQR* Interquartile range, *MCI/AD(* +*)* Participants with aMCI and positive AD biomarkers, *MCI(-)* Participants with aMCI and negative AD biomarkers, *NS* non-significant

#### Proteomic profile associated with GBCA clearance

In the participants from Cohort 1, we evaluated clearance of GBCA from 78 ROIs across the brain. We also evaluated the levels of 7K proteins in their CSF. We identified proteins that were associated with GBCA clearance in all ROIs (at *p*-value < 6.4 × 10^–4^). The number of proteins significantly correlated with GBCA clearance efficiency varied from one ROI to another (from 1 to 65 proteins). In total, seven unique proteins were significantly correlated (*p*-value < 6.4 × 10^–4^) with GS clearance efficiency in at least one ROI (Table 2).

**Table 2:** Proteomic results for the association with the GS clearance efficiency (Stage 1 analysis)

Protein	logFC	Rho	*P*-value	ROI	Number of ROIs
NSUN6	1.098	6.533	4.18E-05	Right Cerebellum White Matter	66
GRAAK	-1.103	-5.804	1.16E-04	Right Transverse Temporal Gyrus	78
NSUN6	0.909	5.688	1.38E-04	Right Lateral Occipital Cortex	66
NSUN6	0.762	5.542	1.72E-04	Right Pericalcarine Cortex	66
GRAAK	-1.167	-5.536	1.74E-04	Right Putamen	78
GRAAK	-1.224	-5.480	1.89E-04	Right Caudate Nucleus	78
SHPS1	-2.140	-5.471	1.92E-04	Right Medial Orbitofrontal Cortex	36
GRAAK	-1.121	-5.360	2.27E-04	Left Superior Frontal Gyrus	78
GRAAK	-1.178	-5.357	2.29E-04	Right Globus Pallidus	78
GRAAK	-0.755	-5.325	2.39E-04	Right Superior Temporal Gyrus	78
GRAAK	-0.941	-5.300	2.49E-04	Right Pars Opercularis	78
NSUN6	0.667	5.261	2.64E-04	Right Middle Temporal Gyrus	66
NSUN6	1.080	5.219	2.81E-04	Right Cerebellar Cortex	66
GRAAK	-0.936	-5.179	3.00E-04	Left Rostral Middle Frontal Gyrus	78
OLFML3	0.400	5.159	3.10E-04	Left Inferior Parietal Lobule	42
GRAAK	-1.181	-5.081	3.49E-04	Right Superior Frontal Gyrus	78
ACTN2	4.623	5.049	3.69E-04	Left Globus Pallidus	68
ACTN2	4.059	5.031	3.77E-04	Left Globus Pallidus	68
GRAAK	-0.689	-5.023	3.83E-04	Right Inferior Temporal Gyrus	78
GRAAK	-0.906	-4.925	4.48E-04	Left Pars Triangularis	78
GRAAK	-0.800	-4.898	4.66E-04	Right Pars Triangulari	78
NSUN6	1.147	4.879	4.81E-04	Left Cerebellum White Matter	66
GRAAK	-1.074	-4.854	5.01E-04	Right Hippocampus	78
GRAAK	-0.584	-4.848	5.06E-04	Right Middle Temporal Gyrus	78
GRAAK	-1.140	-4.832	5.19E-04	Right Insula	78
ACTN2	4.453	4.806	5.43E-04	Right Globus Pallidus	68
GRAAK	-1.114	-4.790	5.56E-04	Left Caudal Middle Frontal Gyrus	78
RUXF	0.415	4.767	5.73E-04	Left Inferior Temporal Gyrus	56
GRAAK	-1.328	-4.750	5.93E-04	Right Thalamus	78
ACTN2	3.537	4.745	6.01E-04	Right Cerebellum White Matter	68
TIM-4	-0.352	-4.732	6.10E-04	Left Medial Orbitofrontal Cortex	35
GRAAK	-1.160	-4.719	6.24E-04	Left Medial Orbitofrontal Cortex	78

The table shows the top associated proteins (FDR significant *p*-values), ROI for which the results is shown and number of associated ROIs

Negative Rho values indicate negative correlation between proteins levels and GS clearance efficiency while positive Rho values indicate positive correlations

Granzyme-Z (GRAAK) was the only protein significantly associated with clearance in all the 78 ROIs (Table 2 and Table S1). The other significant relationships between clearance (in any ROI) and proteins were found for: tRNA (cytosine(72)-C(5))-methyltransferase (NSUN6) significant in 66 ROIs; Tyrosine-protein phosphatase non-receptor type substrate 1 (SHPS1) significant in 36 ROIs; Olfactomedin-like protein 3 (OLFL3) significant in 42 ROIs; Alpha-actinin-2 (ACTN2) significant in 68 ROIs; Small nuclear ribonucleoprotein F (RUXF) significant in 56 ROIs and T cell immunoglobulin and mucin domain containing 4 (TIM-4) significant in 35 ROIs (Table 2).

**Fig. 2:**
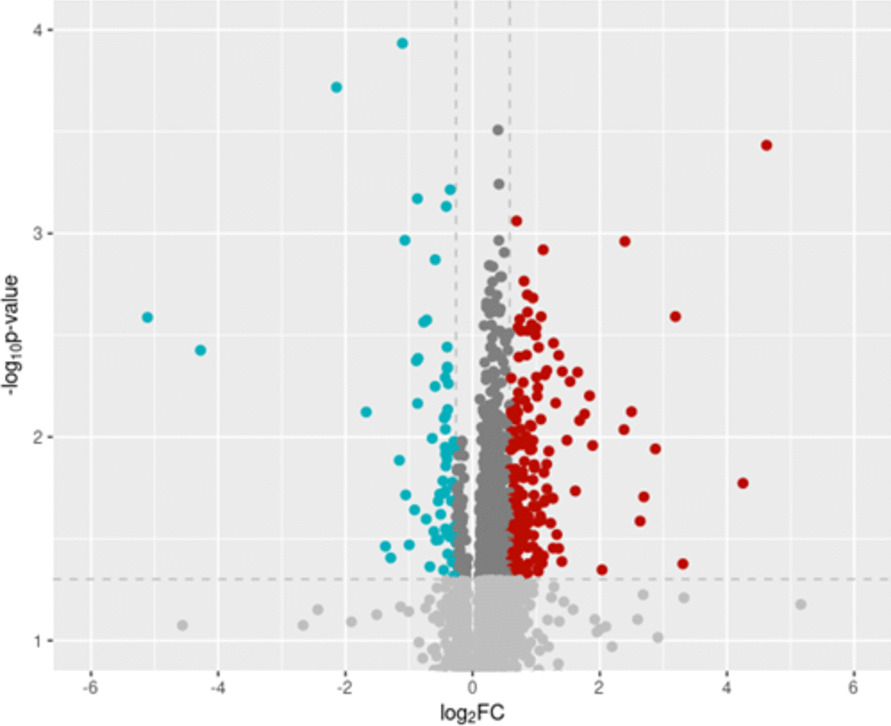
Volcano plot for the “composite” GS clearance efficiency study. The X-axis represents the log2FoldChange (FC) and the Y-axis the -log(*p*-value). Red dots are proteins with significant (*p*-value < 0.05) upregulation (log2FC > 1 standard deviation of the mean) and blue dots are proteins with significant downregulation upregulation (log2FC < 1 standard deviation of the mean)

The majority of relationships between protein level and GCBA clearance were positive correlations (i.e. more efficient GS clearance of GCBA is associated with increased protein presence) (Fig. 2). From the most significant findings, NSUN6, OLFML3, RUXF and ACTN2 showed a positive correlation. Strong negative correlations were observed however for: GRAAK, SHPS1 and TIM-4 (for details of individual proteins see Table 2).

#### Pathway enrichment

We performed a protein set enrichment analysis to identify relevant pathways identified via the analysis above.

**Fig. 3:**
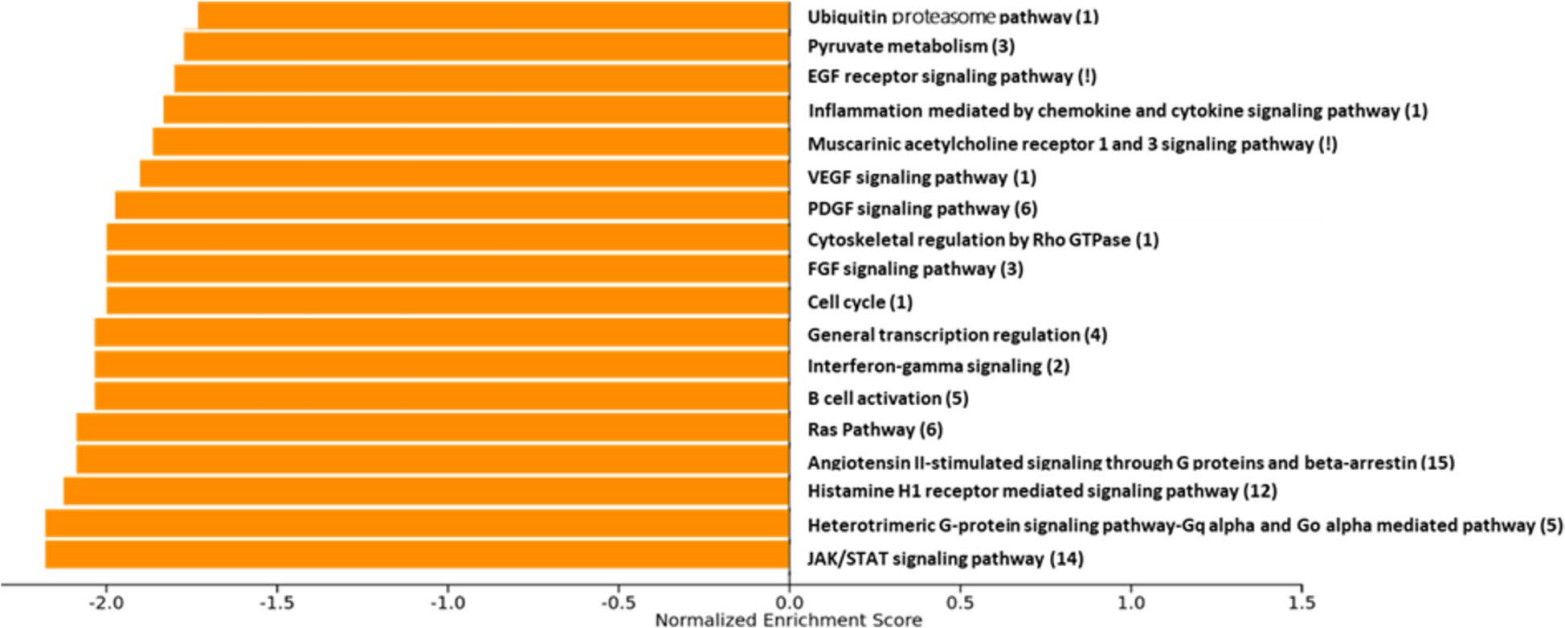
Protein set enrichment analysis from the “single analysis”. The figure shows the significant FDR-corrected pathways associated with GS clearance in some ROI. The number of ROIs in which the pathway is significant is shown in parenthesis after the pathway name

In the “single analysis” and after FDR correction, we identified 18 significant pathways. Some of the pathways were significant only in one ROI while other were commonly significant in different ROIs. The most significant pathway was related with the angiotensin II. Others were involved in inflammation and immunity (Fig. 3).

**Fig. 4:**
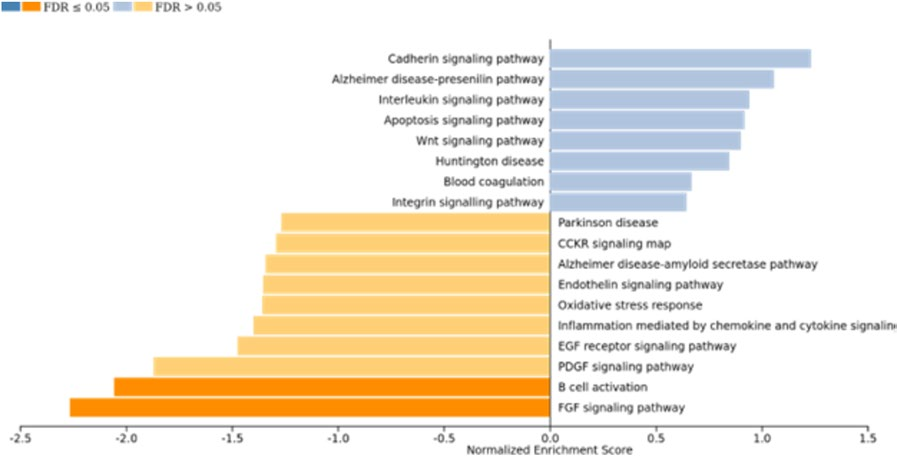
Protein set enrichment analysis with Panther from the “composite analysis”. Pathways with positive (in blue) or negative (in orange) enrichment score in the GS clearance efficiency proteomic dataset

The results from the “composite analysis” showed FDR-corrected significant pathways with a negative enrichment score, indicating that these pathways were enriched with proteins that were downregulated in participants with more efficient clearance of GCBA (Fig. 4). These pathways were FGF signaling and B cell activation (FDR *p*-value = 8.8 × 10^–3^ and 2.4 × 10^–2^, respectively).

Several other pathways were found with a significant (not corrected) *p*-value, which we considered a nominal association. The positive enrichment score indicated an enrichment of upregulated proteins linked with more efficient GCBA clearance (Fig. 4). Identified pathways are linked with neurodegenerative processes: Alzheimer disease-presenilin pathway, Huntington disease, Parkinson disease and Alzheimer disease-amyloid secretase pathway.

### Stage 2

#### Clinical and demographic outcomes

Stage 2 was carried out in a second cohort of participants (*n* = 39): aMCI(-), aMCI/AD( +) and healthy controls. The available clinical and demographic characteristics of these participants can be seen in Table 3.

**Table 3:** Cohort 2 description

		**aMCI/AD**^**a**^ **( +); ** ***n*** **= 7**	**aMCI**^**a**^ **(-); ** ***n*** **= 22**	**Healthy controls; ** ***n*** **= 10**
Demographic	Age; median (SD)	74.14 (3.80)	61.18 (8.05)	73.3 (3.97)
	Sex (% Female)	5(71.4%)	13 (59%)	6 (60%)
Biomarkers (mean)	Amyloid 1:42 (SD)	380 (154)	1404 (285.72)	-
	Tau (SD)	785.14 (187)	219.56 (64.95)	-
Cognition (median)	MMSE (IQR)	20 (7)	29 (3.35)	-

*MMSE* Mini-Mental State Examination

^a^ *MCI/AD(* +*)* Participants with aMCI and positive AD biomarkers, *MCI(-)* Participants with aMCI and negative AD biomarkers, *SD* Standard deviation, *IQR* interquartile range

#### Proteomic vs demographic-only prediction models

With the objective of generating prediction models, first we calculated 78 different scores (one per ROI) based on a maximum of 5 proteins (from the most significant in each ROI) associated with more efficient GCBA clearance in Stage 1.

**Fig. 5:**
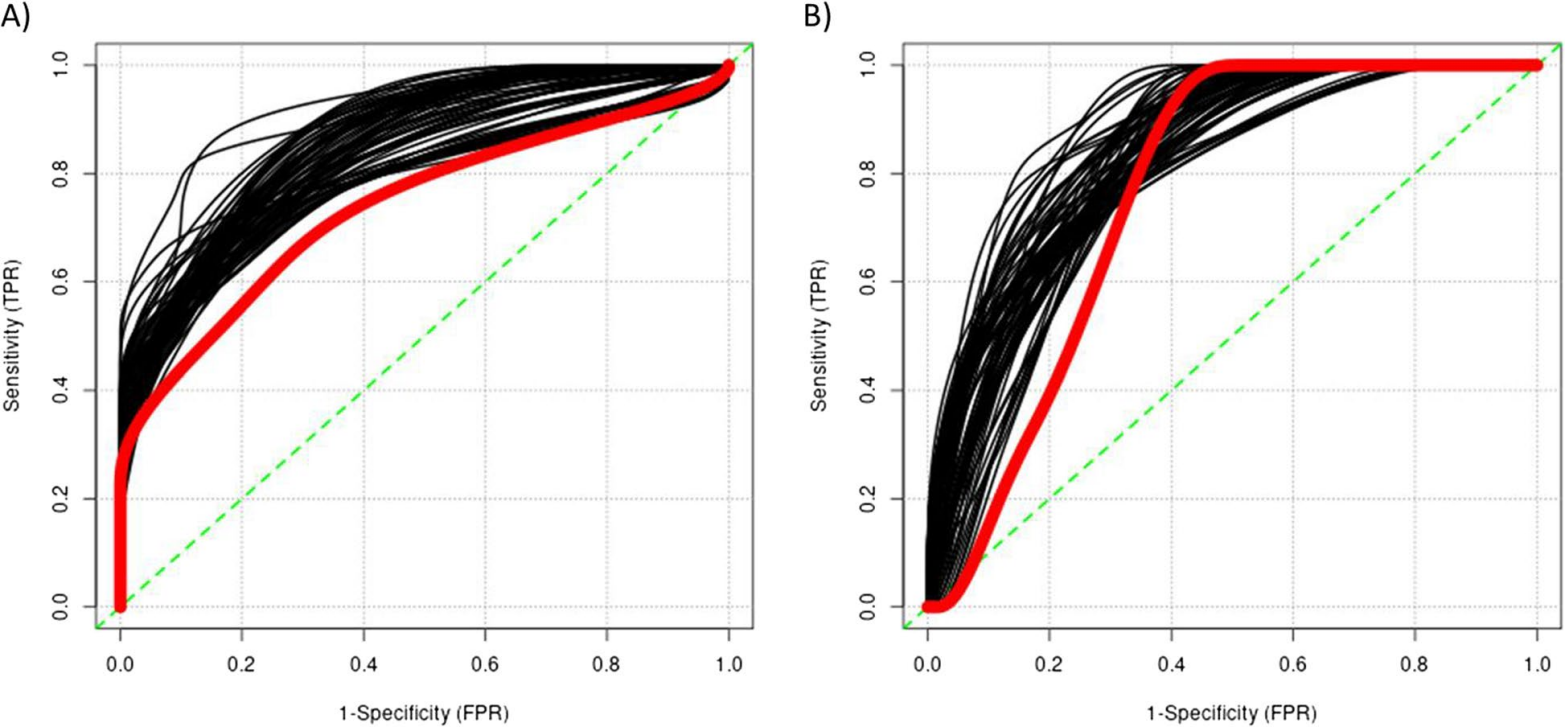
ROC curves for the proteomic ROI-specific (black lines) and demographic (red lines) models. **A** ROC curves for the aMCI prediction and **B** ROC curves for the aMCI/AD( +) prediction

To determine whether proteins identified in Stage 1 and incorporated into proteomic scores can distinguish participants with aMCI (both + and -) from controls, we generated prediction general linear models in the Cohort 2 to determine if inclusion of each score could outperform a demographic-only model. All but eight of the models including a proteomic score outperformed the demographic-only model (AUC from proteomic models range 0.959 to 0.79 vs AUC from demographic-only model = 0.8; Fig. 5A and Table S5). The specificity for each of the proteomic models ranged from 1 to 0.59 and the sensitivity from 0.6 to 1. The demographic-only model predicting aMCI presented a sensitivity of 0.7 and specificity of 0.86.

Similarly, we determined whether the proteomic scores generated from significant proteins in Stage 1 can distinguish participants with aMCI/AD( +) from controls, we followed two approaches. First, following the same approach that in the evaluation of prediction of aMCI (both + and -), we generated prediction general linear models in the Cohort 2 to determine if inclusion of each score could outperform a demographic-only model. All but one of the models including a proteomic score (AUC range 0.99 to 0.81) outperformed the demographic-only model (AUC = 0.81; Fig. 5b and Table S6). The specificity for each of the proteomic models ranged from 1 to 0.57 and the sensitivity from 0.56 to 0.97. The demographic-only model predicting aMCI/AD( +) presented a sensitivity of 0.78 and specificity of 1.

As a second approach, we tested whether a prediction model generated in the Cohort 1 was able to predict participants with aMCI/AD( +) in the Cohort 2. We were not able to do the same for the aMCI(+ and -) vs controls prediction because Cohort 1 did not include healthy controls. A total of 31 proteomic models (AUC range 0.90 to 0.66) generated in the Cohort 1 and tested in the Cohort 2 outperformed the demographic-only model (AUC = 0.81; Table S7). In this analysis, the specificity for each of the proteomic models ranged from 0.85 to 1 and the sensitivity from 0.53 to 0.81. The demographic-only model predicting aMCI/AD( +) tested in the Cohort 2 presented a sensitivity of 0.75 and specificity of 1.

## Discussion

The GS has emerged as a key mediator in the pathogenesis and progression of AD [26]. Both rate and efficiency of clearance of intravenously injected GBCA was identified in aMCI/AD patients [15]. The current study was designed to discover whether proteins expressed in the CSF are associated with GBCA clearance in participants with amnestic mild to moderate cognitive impairment. The findings of this study could have significant clinical implications. It is the first study to quantify GS clearance efficiency combined with robust CSF proteomics study in participants with aMCI and early AD.

We identified seven proteins that were significantly associated with the degree by which GBCA was cleared from the brain after 28 h (GS clearance efficiency) in a wide range of brain ROIs: GRAAK, NSUN6, SHPS1, OLFML3, RUXF, ACTN2 and TIM-4. Remarkably, four of these proteins are relevant in the immune system, emphasizing the role that the immune system has been described to have in the modulation of the GS clearance efficiency [10, 27, 28].

### The role of the immune system in GS clearance efficiency

Our results strongly support immune system involvement in GS regulation, which has been previously linked with GS function [10, 27, 28]. It is well known that both the adaptive and the innate immune cells are implicated in the etiology and pathogenesis of AD [29] and that the immune system is relevant for the function of the GS. In AD, alterations in microglia and peripheral immune cells negatively impact brain function and homeostasis [30]. This proposal is reinforced by evidence that immune cells are hosted in the complex setting of subarachnoid space (SAS) [12, 13]. The border of the SAS has a potential barrier property of the leptomeninges in controlling access of immune mediators and immune cells into the CNS during health and neuroinflammation. This is the first study in humans that potentially links GS health with proteins that are key factors in the immune system regulation.

GRAAK belongs to a family of serine protease proteins stored in granules from immune system’s cytotoxic cells and it is highly expressed in CD8 T cells [31]. It has an important role modulating pro-inflammatory processes and apoptosis [31]. Recently, a single-cell transcriptomic analysis has identified that T cells expressing GRAAK were biomarkers for AD [32]. In a rat model induced with inflammatory injury, an increase in GRAAK expression was identified, which was associated with plaque-like aggregation of myelin, colocalizing with Aβ protein precursor and Aβ [33]. The results from the Multi-omics Atlas Project [34] with information about the expression of different genes in microglia (a key cell type in the GS activity regulation) from AD participants and controls, indicated that *GZMK*, the gene encoding GRAAK protein, presented higher expression in microglia from AD than from controls. These results are in accordance with our results and suggest that the higher expression of this protein is linked with poor GS clearance efficiency and increases the risk of AD.

SHPS1 is a member of the signal regulatory protein family. Several functional roles attributed to this protein are related with AD pathology and GS function: neuronal survival, synaptogenesis, entrainment of circadian clock and negative regulation of immune system cells [35]. SHPS1 has been previously related with AD through the function of its ligand, CD47, which is expressed in astrocytes and neurons. It has been described that CD47, mediated by SHPS1, regulates the expression of proinflammatory cytokines [36].

ACTN2 is a cytoskeletal protein, involved in the actin binding to the membrane. In a study analyzing by single nuclei transcriptomics the expression profile of AD, ACTN2 was one of the genes characterizing specific clusters of oligodendrocytes in AD [37]. Considering the role of oligodendrocytes in the immune system, our results suggest that ACTN2 could be mediating GS clearance changes by acting in immune pathways [38].

TIM-4 was also identified as a key regulator of GS function in our results. TIM‑4 is expressed in immune cells, mainly in T cells but also B and mast cells and participates in multiple aspects of immune regulation [39]. It has been previously associated with AD risk. In a large Genome-Wide Association Study (GWAS) in people with AD, TIM-4 was mapped to one of the significant loci associated with AD, reinforcing the relevance of TIM-4 in AD [40].

The other three proteins (NSUN6, OLFML3 and RUXF) do not belong to the immune pathway but they have been previously related with AD. NSUN6 has been identified to be downregulated in brain from AD participants compared to controls [41]. OLFM3 has been found to be expressed in amyloid plaques from AD patients [42] and RUXF was classified among the top 10 hub shared between AD and type 2 diabetes [43].

### Relevant pathways in GS clearance regulation

Proteomics studies are useful for biomarker identification and can help discover pathways relevant to disease. The link between immune responses and GS efficiency in the early stages of AD described here underlies that these pathways maybe important to disease. Given that GS is modifiable, identifying pathways associated is of particular interest.

Our “composite analysis” points to two significant pathways: B cell activation and the FGF pathway. B cell activation is a key process in the immune system and has been previously related to AD risk through antibody dependent and independent mechanisms [44]. Some B-cell relevant functions related to AD are the antibody presentation against aβ deposition and the regulation of inflammation by cytokines [44]. The FGF pathway is involved in key processes such as cell survival, proliferation, tissue repair, and metabolism [45].

Further, we identified different pathways related with neurodegenerative diseases: we found an enrichment of proteins related with GS clearance efficiency that were key mediators for the development of these diseases. To highlight, we recognized AD-specific pathways. The AD-presenilin pathway had a positive enrichment score, including Frizzled proteins (FZD1,2,4,7 and 9). These proteins are receptors involved in the Wnt pathways (nominally associated in our pathway enrichment results). They have been previously linked to AD through their role in the assembly of functional neuronal circuits which are relevant in cognition [46]*.* FZD proteins were positively associated with GS clearance efficiency, which indicates that higher levels of the proteins were linked with increased clearance efficiency. These findings correlate with the crucial function of these proteins previously described in axon guidance, dendritogenesis, and synapse formation [47]. In contrast, the AD- amyloid secretase pathway was found to be negative enriched. The main proteins identified were MAPK10, PRKCI and PRKCQ: all of them with a negative association with GS clearance efficiency (proteins had lower expression in association with greater GS clearance). Interestingly, MAPK10 was previously described to be increased in AD participants [47].

The protein pathway enrichment analysis from the “single analysis” further points to the immune system (mainly through B cell activation) and inflammatory processes via different pathways (histamine regulation, angiotensin II, JAK/stat, RAS, among others) as a key mediators linked to GS clearance efficiency in participants with risk of AD. Other pathways include angiogenesis (through VEG regulation), cytoskeleton regulation, oxidative stress and apoptosis. Many of these pathways have been previously related with AD risk [29]. For instance, the role of apoptosis has been extensively described in AD. It may represent one of the stages of AD because of amyloid accumulation, inflammation, or mitochondrial dysfunction. The activation of apoptosis in AD occurs in an abnormal form which affects cellular organelles and leads to the progression of AD [48]. Moreover, AQP4, a key element of the GS is also involved in neuronal apoptosis [49].

The cytoskeletal regulation was also identified to be associated with clearance efficiency. It is the main component of the actin cytoskeleton. Cytoskeletal dynamics dysregulation is a common feature of neurodegenerative diseases, including AD [50]. The actin is involved in the formation of dendrites and synapsis, and it has been previously implicated in AD. In a recent proteomic study, the same pathway was found to be associated with resilience to AD, defined as a combination of high disease burden without dementia [51]. In a previous study, which analyzed the effect of a neuroprotective drugs in a cell model of AD, the actin filament pathway was also identified to be involved in the response to the drug treatment [52]. Our results, combined with the previously published results, could potentially implicate proteins related to actin filament regulation in GS activity that could be relevant for AD progression.

### Clinical implications

The findings of this study could have significant clinical implications. To our knowledge, this is the first study to quantify GS clearance efficiency combined with robust CSF proteomics study in participants with aMCI and early AD. The models that we generated with proteomics data were associated with clearance and demonstrated good discrimination for aMCI. This points that GS clearance efficiency is a relevant biomarker to determine the risk and the progression of AD and that targeting the GS may directly impact AD risk and prognosis. As a future step, it would be relevant to determine whether the modulation of these proteins is a cause or consequence of GS regulation by using approaches such as Mendelian Randomization. If causal relationship between any of the current associations and AD is identified, it could serve as a starting point for developing potential drug targets for these proteins.

To increase the predictable value of our findings, it would be relevant to include data of the blood–brain barrier (BBB) integrity because of its implication in brain clearance processes. The dysregulation of the BBB has been previously linked with cognition [53–55] and together with GBCA data may better describe clearance dysfunction in subjects with MCI and early AD.

The study main limitation is the small sample size which could limit the generalizability of the current findings. Although further studies with increased sample size are needed to validate the results, the identification of AD related pathways in the enrichment analyses indicate enough power to detect relevant proteins for the GS system. It is relevant that for the first-time proteomic biomarkers have been studied and linked with GS health. Also, it is the first study in which the GS efficiency in aMCI has been evaluated using an intravenous contrast agent [15].

## Conclusions

We point to seven proteins as key molecules linking altered immune responses in aged participants with failure in GS function. These proteins linked with GS function could be good biomarkers for the prediction of MCI and AD and indicate that targeting GS could help to modulate AD progression. Molecular studies with in vitro and in vivo analyses could help to disentangle the mechanism by which these proteins and the GS regulates the AD progression.

## Supplementary Information

Supplementary Material 1.

## Data Availability

The datasets analysed during the current study are available from the corresponding author on reasonable request.

## Abbreviations

GS: Glymphatic system
AD: Alzheimer’s disease
GBCA: Gadolinium-based contrast agent
MRI: Magnetic resonance imaging
aMCI: Mild cognitive impairment
ROIs: Brain regions of interest
AQP: Water channel aquaporin
CSF: Cerebrospinal fluid
RBANS: Repeatable Battery for the Assessment of Neuropsychological Status
MMSE: Mini-mental state examination
CDR: Clinical Dementia Rating
p-tau: Phosphorylated tau at threonine 181
RFU: Relative fluorescence units
GSEA: Gene Set Enrichment Analysis
Glm: General linear model
GRAAK: Granzyme-Z
NSUN6: TRNA cytosine72-C5-methyltransferase
SHPS1: Tyrosine-protein phosphatase non-receptor type substrate 1
OLFL3: Olfactomedin-like protein 3
ACTN2: Alpha-actinin-2
RUXF: Small nuclear ribonucleoprotein F
TIM-4: Metalloproteinase inhibitor 4
SAS: Subarachnoid space
FZD: Frizzled proteins
BBB: Blood–Brain Barrier
